# Supplementary material for: A Kirkpatrick Model Process Evaluation of Reactions and Learning from My Strengths Training for Life™
Source: Int J Environ Res Public Health. 2022 Sep 8;19(18):11320. doi: 10.3390/ijerph191811320 (PMC9517080; doi:10.3390/ijerph191811320)
Supplement: Supplementary file 1 [file ijerph-19-11320-s001.zip › ijerph-1897452-supplementary.pdf]

Supplement Table S1 *Preliminary Analyses for Demographic Differences in Reaction and Learning Variables*

	Attendance	Engagement	Program evaluation	Facilitator evaluation	Program enjoyment	Transfer intention	Mental skills
Gender	$F(3, 270) = .58$, $p = .626$, $\eta_p^2 = .01$, observed power = 17%	$F(3, 270) = 1.42$, $p = .238$, $\eta_p^2 = .02$, observed power = 37%	$F(3, 104) = 1.35$, $p = .262$, $\eta_p^2 = .04$, observed power = 35%	$F(3, 104) = 1.39$, $p = .252$, $\eta_p^2 = .04$, observed power = 36%	$F(3, 104) = .56$, $p = .640$, $\eta_p^2 = .02$, observed power = 16%	$F(3, 99) = .59$, $p = .625$, $\eta_p^2 = .02$, observed power = 17%	Pillai's Trace = .12, $F(18, 276) = .60$, $p = .900$, $\eta_p^2 = .04$, observed power = 43%
Ethnicity	$F(5, 261) = .89$, $p = .491$, $\eta_p^2 = .02$, observed power = 32%	$F(5, 260) = 1.56$, $p = .172$, $\eta_p^2 = .03$, observed power = 54%	$F(4, 94) = .60$, $p = .665$, $\eta_p^2 = .03$, observed power = 19%	$F(4, 94) = .13$, $p = .973$, $\eta_p^2 = .01$, observed power = 8%	$F(4, 94) = .49$, $p = .746$, $\eta_p^2 = .02$, observed power = 16%	$F(4, 90) = 1.11$, $p = .357$, $\eta_p^2 = .05$, observed power = 34%	Pillai's Trace = .15, $F(24, 336) = .56$, $p = .956$, $\eta_p^2 = .04$, observed power = 48%
Social inclusion	$F(3, 249) = .89$, $p = .897$, $\eta_p^2 = .01$, observed power = 8%	$F(3, 249) = 3.11$, $p = .027$*, $\eta_p^2 = .04$, observed power = 72%	$F(3, 87) = 2.12$, $p = .104$, $\eta_p^2 = .07$, observed power = 52%	$F(3, 87) = 1.27$, $p = .289$, $\eta_p^2 = .04$, observed power = 33%	$F(3, 87) = 3.13$, $p = .030$*, $\eta_p^2 = .10$, observed power = 71%	$F(3, 83) = 2.33$, $p = .080$, $\eta_p^2 = .08$, observed power = 57%	Pillai's Trace = .30, $F(18, 231) = 1.42$, $p = .124$, $\eta_p^2 = .10$, observed power = 88%
Learning difficulty	$F(2, 193) = 3.10$, $p = .047$*, $\eta_p^2 = .03$, observed power = 59%	$F(2, 193) = .81$, $p = .447$, $\eta_p^2 = .01$, observed power = 19%	$F(2, 63) = .17$, $p = .848$, $\eta_p^2 = .01$, observed power = 7%	$F(2, 63) = .47$, $p = .625$, $\eta_p^2 = .02$, observed power = 12%	$F(2, 63) = 1.75$, $p = .183$, $\eta_p^2 = .05$, observed power = 35%	$F(2, 61) = 1.44$, $p = .246$, $\eta_p^2 = .05$, observed power = 30%	Pillai's Trace = .18, $F(12, 110) = .89$, $p = .564$, $\eta_p^2 = .09$, observed power = 49%

Note. *Became non-significant after Benjamini-Hochberg correction.
